# Ionic Conductive Gels for Optically Manipulatable Microwave Stealth Structures

**DOI:** 10.1002/advs.201902162

**Published:** 2019-11-27

**Authors:** Wei‐Li Song, Ya‐Jing Zhang, Kai‐Lun Zhang, Ke Wang, Lu Zhang, Li‐Li Chen, Yixing Huang, Mingji Chen, Hongshuai Lei, Haosen Chen, Daining Fang

**Affiliations:** ^1^ Institute of Advanced Structure Technology Beijing Institute of Technology Beijing 100081 P. R. China; ^2^ Beijing Key Laboratory of Lightweight Multi‐Functional Composite Materials and Structures Beijing Institute of Technology Beijing 100081 P. R. China; ^3^ School of Materials Science and Engineering Beijing Institute of Technology Beijing 100081 P. R. China; ^4^ Key Laboratory of Space Utilization Technology and Engineering Center for space Utilization Chinese Academy of Sciences Beijing 100094 China

**Keywords:** dielectric materials, gels, optical structures, smart windows, transparent microwave absorbers

## Abstract

Smart structures with manipulatable properties are highly demanded in many fields. However, there is a critical challenge in the pursuit of transparent windows that allow optical waves (wavelength of µm–nm) for transmitting while blocking microwave (wavelength of cm) in terms of absorbing electromagnetic energy, specifically for meeting the frequency requirement for the 5th generation (5G) mobile networks. For fundamentally establishing novel manipulatable microwave absorbing structures, here, new polymeric aqueous gels as both optically transparent materials and microwave absorbing materials are demonstrated, in which polar networks play significant roles in attenuating electromagnetic energy. By manipulating the hydrogen bonding networks, the resulting optically transparent solid‐state gels are able to offer the capabilities for absorbing microwaves. Interestingly, such gels can be switched into an optically opaque state via converting the amorphous state into a polycrystal state when the temperature is decreased. Such ionic conductive gels can endow the assembled sandwich windows with effective microwave absorbing capability in the range of 15–40 GHz, covering a branch of 5G frequency bands. The results highlight a new strategy for using ionic conductive gels to design and fabricate manipulatable microwave stealth structures for various applications.

## Graphical abstract

Novel transparent ionic conductive gels are developed to fabricate optically manipulatable microwave stealth structures, which can be employed as transparent windows while blocking microwaves in terms of absorbing electromagnetic energy. Such smart gels can endow the assembled windows with effective microwave absorbing capability in the range of 15–40 GHz, advancing smart windows with upgraded manipulatable capabilities and functions.

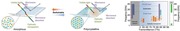

## 1 Introduction

**Figure 1:**
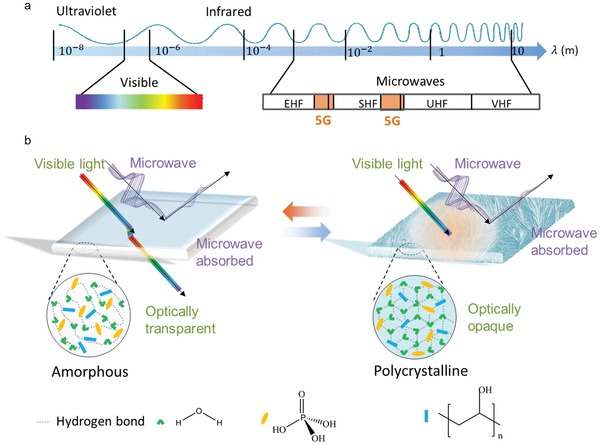
Switchable smart microwave stealth structures: a) Division of electromagnetic wave bands as well as communication bands for the 5G mobile networks. b) Schematic of optically transparent–opaque switchable smart microwave stealth structures in this study.

Optically transparent structures and windows are increasingly attractive because they possess wide applications in a variety of industries for their unique functions. With developing smart technologies, smart windows and structures are highly pursued because they are expected to offer exclusive variable function when environment condition changes.^1^, ^2^, ^3^, ^4^, ^5^, ^6^, ^7^, ^8^ In the past decades, advanced technology has been carried out to the smart optical windows with capabilities of varying color or blocking lights. In a typical smart window with switchable feature, external stimuli, such as mechanical force, thermal energy, electricity, and chemical molecules, could induce the change of material properties or geometric parameters of the materials and windows.^9^, ^10^ In the range of electromagnetic waves (**Figure** **1**a), visible waves with nm wavelength are sensitive to the materials in the nanoscale size or with an appropriate bandgap.^11^ In the microwave range, the electromagnetic waves with cm wavelength are widely used for telecommunication. For instance, telecommunication technology within super high frequency (SHF: 3–30 GHz) and extremely high frequency (30–300 GHz) is intensively explored for meeting the requirements of 5th generation (5G) mobile networks (operation frequency bands: branch I: 0.45–6 GHz; branch II: 24.25–40 GHz or higher) (Figure 1a). With telecommunication development, the utilization of 5G technology would induce health concerns mainly from enlarged frequency with enlarged energy and electromagnetic energy density. For alleviating the concerns, a variety of novel multifunctional electromagnetic responsive materials and structures, such as coatings, windows, and devices, are expected to be substantially developed.

In addition, optically transparent windows with microwave absorption capability is another significant technique in many fields, including detection, scanning, and communication technologies. Nonetheless, the traditional microwave absorption materials in SHF with capabilities in either electrical loss or magnetic loss are generally in an optically opaque fashion.^12^, ^13^, ^14^, ^15^, ^16^, ^17^, ^18^, ^19^, ^20^, ^21^, ^22^, ^23^, ^24^, ^25^, ^26^, ^27^ Apparently, there are plenty of efforts should be drawn for designing and processing those traditional microwave absorption materials into an optically transparent configuration. For addressing such bottlenecks, attempts have been made by utilizing the electrically conductive transparent thin films to design optically transparent structures.^1^, ^2^, ^3^, ^4^ In a typical examples, Jang et al. reported a metal aluminum mesh to fabricate a wave reflection pattern and polydimethylsiloxane is used as a transparent medium for electromagnetic wave loss.^1^ In the study by Lee et al.^4^ an indium tin oxide (ITO) conductive sheet was used as a reflective plate, followed by assembling with a conductive metal mesh pattern as a frequency selective surface. The structure presented a light transmittance –86% (at 550 nm) as well as 4.28 GHz in the effective bandwidth (the bandwidth of reflection loss ←10 dB). According to the state‐of‐the‐art of transparent microwave absorption structures,^1^, ^2^, ^3^, ^4^ the active materials for manipulating the microwaves are also electrically conductive and they have certain capabilities in absorbing optical lights due to their intrinsic conductive features. It is an urgent to develop novel prototypes of materials that enable to absorb microwave,^18^, ^19^, ^28^, ^29^, ^30^ but allow optical light for transmitting.

For overcoming the critical challenge of such smart transparent windows as well as meeting the required frequency bands of the new telecommunication protocols established on the 5G mobile networks, in this contribution, we developed aqueous gels as a novel type of optically transparent–opaque switchable smart electromagnetic absorbing material for constructing optically transparent–opaque switchable microwave stealth windows. Such gels with temperature‐dependent crystalline structures were able to vary the intrinsical optical properties, with the results of allowing the optical lights for either transmitting or scattering. By designing the sandwich structure windows, the assembled window can deliver broadband effective microwave absorption properties within the frequency range of up to 15–40 GHz, which cover a branch of the 5G frequency band (24.25–40 GHz). Compared with previous developed smart windows and transparent microwave absorption structures, the smart windows upon the aqueous gels here hold the both advantages for offering enhanced functions to advance window performance.

## 2 Results

In the design of the smart windows (Figure 1b), an optically transparent poly(methyl methacrylate) (PMMA) sandwich structure was employed as the framework and an aqueous poly(vinyl alcohol) (PVA)‐based gel was prepared as the active materials for both microwave absorption and optically transparent–opaque switch. At the bottom, an optically transparent and electrically conductive ITO substrate serves as the microwave reflection plate. Apparently, the key component in the smart structure is the aqueous PVA‐based gel. For fabricating a favorable gel with effective microwave absorption properties, various types of aqueous PVA‐based gels were initially tailored with different components (**Table** **1**), followed by selecting the favorable candidate for further structure design (Figure 1b). For the mechanism of the microwave absorption from aqueous PVA‐based gels, the highly polar water molecules play a critical role in attenuating the electromagnetic waves in terms of dielectric loss.^31^, ^32^, ^33^ Meanwhile, addition of PVA polymers with hydroxyl functional groups and phosphoric acid molecules is responsible for tailoring the mobility of water molecules in forms of creating crosslinking hydrogen‐bonding network. For the mechanism of the optically transparent–opaque switchable behaviors, all the materials used for constructing the structures are in amorphous fashion and they are all optically transparent at room temperature. When the temperature is decreased to subzero temperature, the aqueous PVA‐based gels would become polycrystalline, leading to form optically opaque gels for their ability in scattering optical waves. Interestingly, it is noted that such optical feature is easily reversible because the aqueous PVA‐based gels possess temperature‐dependent polycrystal–amorphous switchable ability.

**Table 1:** Different component ratios of various prepared gel materials

		H_2_O [wt%]	H_3_PO_4_ [wt%]	PVA [wt%]
Neat water	H_2_O	100	0	0
Sample 1	10%PVA	90	0	10
Sample 2	12.5%PVA	87.5	0	12.5
Sample 3	15%PVA	85	0	15
Sample 4	10%PVA–5%H_3_PO_4_	85	5	10
Sample 5	10%PVA–12%H_3_PO_4_	78	12	10

**Figure 2:**
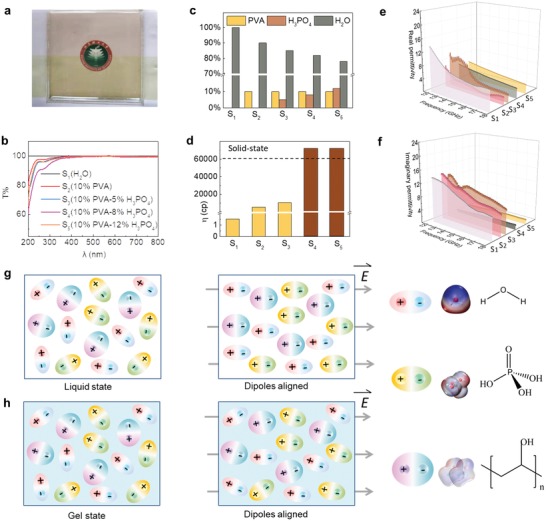
Intrinsical materials properties of the developed gels: a) The photo of the assembled smart microwave stealth structure, in which a single‐layer gel was embedded. b) Optical transmittance of various gel samples in the wavelength of 200–800 nm. c) Component content, d) viscosity, e) real permittivity, and f) imaginary permittivity of various gel samples. g,h) Polarization schemes of various polar molecules in the liquid state (g) and gel state (h) within/without the electromagnetic field.

In the assembly of the smart microwave stealth structures (**Figure** **2**a), the aqueous PVA‐based gels were initially prepared. In the typical preparation, various amounts of PVA and phosphoric acid were mixed in the sealed hot aqueous solutions, and the loadings of each component were illustrated in Figure 2c. Upon stirring the mixture into a transparent state and cooling to room temperature, the as‐prepared six types of aqueous PVA‐based gels were characterized by various technologies. According to the UV–vis spectra (Figure 2b), it is clear that all the aqueous PVA‐based gels are highly transparent in the most range of visible light. As exhibited in Figure 2d, the results of viscosity measurement suggest that there are two types of aqueous PVA‐based gels, i.e., solid state (samples 4 and 5) and liquid state (samples 1, 2, and 3). The solid‐state gel could be understood that the phosphoric acid would be an addictive of crosslinker to stabilize the aqueous PVA gels in terms of generating hydrogen‐bonding networks.

In the determination of the complex permittivity, a polymeric chamber with dimension of 15.8 mm × 7.9 mm × 4 mm was 3D printed with VeroWhite resin, according to our previous studies.^30^ Subsequently, the aqueous PVA‐based gels were transferred into the chamber to achieve complex permittivity in the Ku band (12.4–18 GHz). Since the complex permittivity of the thin‐walled chamber printed by VeroWhite resin is similar to air, the measured values are almost attributed to the aqueous PVA‐based gels. Apparently, the solid‐state samples exhibit completely different curves in comparison with the liquid ones. As plotted in Figure 2e,f (more details in Figure S1, Supporting Information), both the real and imaginary parts of solid‐state samples present much more smooth curves. It is easy to understand that the polar molecules in the aqueous PVA‐based gels would generate polarization under electromagnetic field, and thus pristine water (full of polar molecules) would exhibit the highest polarization in the investigated range. More importantly, the imaginary parts of the solid‐state samples are much lower that the liquid ones, which indicates that the polar molecules in the liquid phase would be more sensitive to electromagnetic waves and would generate enhanced dielectric loss in terms of molecule displacement.^31^, ^32^, ^33^ Alternatively, the polar molecules in the solid‐state samples with more robust hydrogen‐bonding networks would be more stable, and therefore the polarized capabilities would be suppressed under the electromagnetic fields. As a result, both the real and imaginary permittivity of the solid‐state gels were found to be largely decreased (Figure 2g,h).^23^, ^32^, ^33^

**Figure 3:**
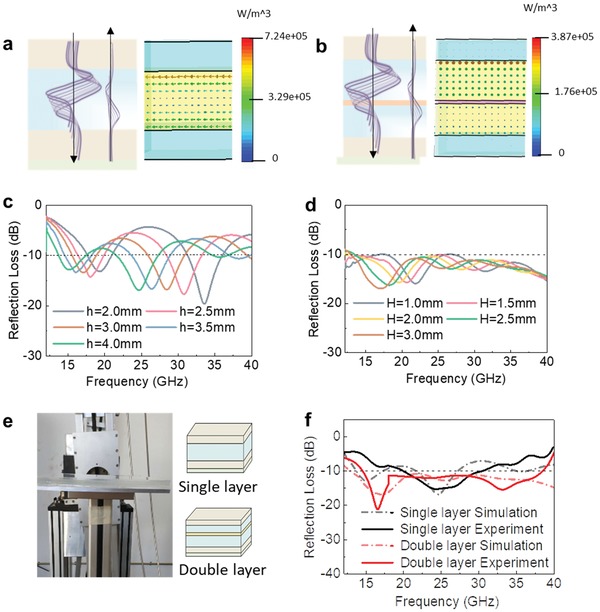
Microwave absorption performance of the designed single layer and double layer sandwich structures: a,b) Power loss density results in CST simulation of the single layer sandwich structure (a) and double layer sandwich structure (b). c) CST‐simulated single layer sandwich structure with different gel thicknesses. d) CST‐simulated double layer sandwich structure with different gel thicknesses. e) Experimental measurement of the microwave absorption performance using the arch method, along with the schemes for the designed two types of window structures. f) Comparison of simulated and experimental microwave performance of the single layer and double layer sandwich structures.

According to the measured complex permittivity, the calculated reflection loss based on a layer of gel coated on the perfect electrical conductor plane was obtained, as shown in Figure S2 in the Supporting Information. Apparently, the sample 5 (10% PVA–12% H_3_PO_4_) with more enhanced microwave absorption capabilities should be selected for designing transparent stealth window structures. In the design of the structures using the Computer Simulation Technology (CST) software, the complex permittivity of all the materials were input into two sandwich structure models, i.e., a single layer sandwich structure and a double layer sandwich structures (**Figure** **3**a,b). According to the design of the single sandwich structure, there are four stacked parts. From the top to the bottom, they are PMMA cover (thickness: 2 mm), active PVA gel (thickness: h), PMMA bottom (thickness: 2 mm), and ITO reflection substrate (thickness: 0.5 mm). In the design of the double layer sandwich structure, there are six stacked parts. From the top to the bottom, they are PMMA cover (thickness: 2 mm), the 1st active PVA gel (thickness: H), poly(ethylene terephthalate) (PET) interlayer (thickness: 0.5 mm), the 2nd active PVA gel (thickness 3 mm), PMMA bottom (thickness: 2 mm), and ITO reflection substrate (thickness: 0.5 mm). Additionally, the electromagnetic diagrams for power loss density in two sandwich structures were provided in Figure 3a,b. By tailoring the thickness of the PVA‐based gel, a series of simulated reflection loss curves for the single layer and double layer sandwich structures were plotted in Figure 3c,d, respectively. Comparison of the simulated results suggests that the transparent window with double layer sandwich structures would hold more greater performance in microwave absorption, with wider effective microwave absorption bandwidth. According to the design results, the multilayer structures with gel thicknesses of *h* = 4 mm and *H* = 4 mm were selected for assembling the smart windows of single layer sandwich structures and two layer sandwich structures, respectively.

Based on the design of smart microwave stealth structures, we have further fabricated the structures using PMMA frames and ITO substrates, followed by sealing the as‐prepared PVA‐based gel (Figure 2a). The practical reflection loss performance of the as‐fabricated two structures was experimentally measured using arch method (Figure 3e), and the simulated and measured results are plotted in Figure 3f. The comparison suggests that the as‐fabricated optically transparent window structures present similar curve to the simulated ones.

**Figure 4:**
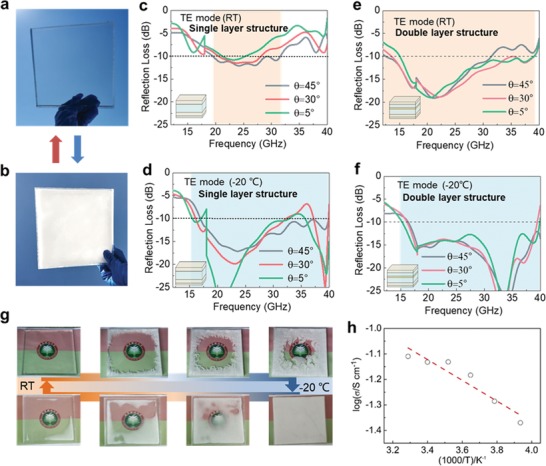
Optically transparent–opaque switching and microwave absorption abilities: a,b) Photographs for the window of the double layer sandwich structure at room temperature (a) and at −20 °C (b). c–f) Experimental microwave absorption performance (transverse electric (TE) mode) achieved by the arch method: single layer sandwich structure at room temperature (c) and at −20 °C (d), as well as double layer sandwich structure at room temperature (e) and at −20 °C (f). The incident angles were set at 5°, 30°, and 45°. g) Demonstration of the optically transparent–opaque switching for the assembled window of double layer sandwich structure, under a temperature cycle from room temperature to −20 °C followed by heating back to room temperature. h) Ionic conductivity of the gels with a temperature of −20–30 °C.

For demonstrating the smartness in optically transparent–opaque switch ability, the as‐fabricated microwave stealth structure was positioned under different environmental temperatures. Initially, the smart structure is expected to be optically transparent at room temperature for the amorphous feature in the aqueous PVA‐based gel (**Figure** **4**a). When the environment temperature was decreased to −20 °C, the smart structure was converted into an optically opaque feature owing to the formation of polycrystal gels in the subzero environment (Figure 4b). Meanwhile, the corresponding microwave absorption performance under different temperature conditions was also experimentally acquired using the arch method. Compared to the microwave absorption performance at room temperature (Figure 4c–f and Figure S3, Supporting Information), the decreased temperature in the subzero environment would slightly change the effective microwave absorption bandwidth of the optically opaque structure, which is responsible for the variation of materials features under different temperature. Nonetheless, both the broadband microwave absorption features in either optically transparent or opaque state would be well maintained, enabling for effectively attenuating electromagnetic waves under different environments. In the designed double layer sandwich structure, the bandwidth for effective microwave absorption was in the frequency range of 15–40 GHz, which enables to cover one of the branch of the 5G frequency bands (24.25–40 GHz). For confirming the reversibility of optically transparent–opaque switching, the structure was repeatedly cooled to −20 °C and heated back to room temperature, as shown in Figure 4g. As anticipation, the optically transparent and opaque features could be well realized when the environment temperature changed (Figure 4g).

## 3 Discussion

**Figure 5:**
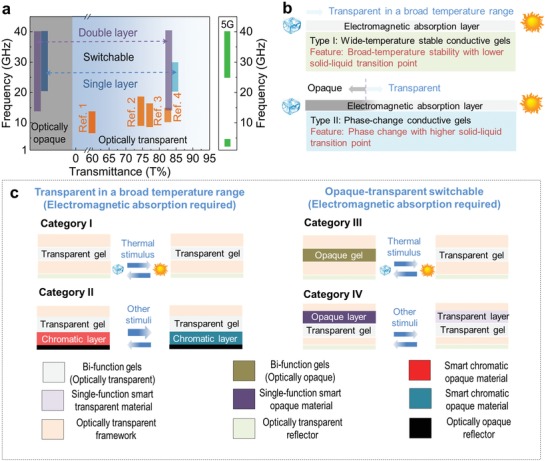
The state‐of‐the‐art and perspectives in multifunction optically manipulatable structures: a) The state‐of‐the‐art of transparent structures for microwave absorption in the GHz range: Comparison of studies related to optically transparent materials, along with the frequency branches for the 5G mobile networks. b) Two representative polymeric gels with microwave absorption capability: Type I as wide‐temperature stable ionic conductive polymeric gels established by lower solid–liquid transition point (even below the application temperature range) and Type II as optically transparent–opaque switchable ionic conductive gels with higher solid–liquid transition point (within the application temperature range). c) Perspective of optically manipulatable microwave stealth structures based on ionic conductive gels and four categories of optically manipulatable microwave stealth structures could be envisaged, specifically by integrating with other types of optical functional structure and devices. Category I as broad‐temperature stable optically transparent microwave stealth structures. Category II as the multifunctional chromatic structure integrated with other active chromatic layers (triggered with other forms of energy) while the above microwave stealth cover remains optically transparent. Category III as the optically transparent–opaque switchable microwave stealth structure, in which the gels are thermally responsive as both optically switchable and microwave absorption materials. Category IV as the multifunctional structure integrated with another type of optically active layer (triggered with other forms of energy) for smart optical structures. (Noted that fabrication of the multilayered structure from all the categories are required additional design and fabricated using the redetermined material parameters.)

For illustrating the advantages in the present study, the state‐of‐the‐art of transparent structures for microwave absorption in the GHz range (**Figure** **5**a) as well as the smart windows in recent studies (Table S1, Supporting Information) are provided. According to the comparison, it is implied that the structures and devices in Table S1 in the Supporting Information could deliver a single function in either microwave absorption^1^, ^2^, ^3^, ^4^ or optical property manipulation.^34^, ^35^, ^36^, ^37^, ^38^, ^39^, ^40^, ^41^, ^42^, ^43^ However, the smart structures upon aqueous PVA‐based gels here could simultaneously hold both features (Table S1, Supporting Information), not only in broadband microwave absorption but also in optical property manipulation without significantly impacting the microwave absorption.

The results suggest that the developed smart microwave stealth windows apparently highlight several unique features beyond the conventional structures and devices. Initially, there is a new strategy for manipulating complex permittivity. The intrinsic complex permittivity of the new gel materials would be tuned with different approaches. In the aqueous solution, polar water molecules are the essential factors for generating polarization and dielectric loss (Figure 2e,f). Aiming to manipulating the polar molecules, addition of PVA gels and phosphoric acid plays as the role in creating crosslink networks and tailoring the hydrogen bonding, respectively. As a consequence, both the polarization and dielectric loss have been well suppressed, leading to stabilizing the complex permittivity. Additionally, to verify the clarification of loss from ions, the ionic conductivity measurement was carried out in the temperature of −20–30 °C as shown in Figure 4h. The results suggest that the values of ionic conductivity of the gels were all on the order of 10^−1^ S cm^−1^, which is sufficiently conductive in a broad temperature range.

Second, optically transparent polymeric gels here open a novel platform for microwave absorption materials and structures. As is well known, microwave absorption materials are usually optically opaque dielectric materials with ability to attenuate electromagnetic energy in terms of converting into electrical and thermal energy.^18^, ^19^, ^28^, ^29^, ^30^ In the previous studies for achieving optically transparent microwave absorption, electrical conductive materials, such as ITO and metals, were processed into electrically conductive patterns, serving as the effective materials for electromagnetic attenuation. In the present study, however, ITO was solely served as the electrical reflection substrate for creating quarter‐wavelength resonance interference,^2^ while aqueous PVA‐based gels are the key effective materials for attenuating electromagnetic energy. Therefore, such concept offers a novel platform to construct various types of microwave absorption structures for stealth windows (Figure 5b), Type I as wide‐temperature stable ionic conductive polymeric gels established by lower solid–liquid transition point (even below the application temperature range) and Type II as optically transparent–opaque switchable ionic conductive gels with higher solid–liquid transition point (within the application temperature range). In a typical example of type I ionic conductive polymeric gels, large amounts of ionic liquids as well as the organic solvents with lower melting points could be used to fabricate broad‐temperature ionic conductive gels. For instant, the optical transparent gels could be achieved by mixing 1‐ethyl‐3‐methylimidazolium chloride ([EMIm]Cl) with aluminum chloride (AlCl_3_) 1:1.3 in molar ratio, followed by polymerization with acrylamide monomers, according to our previous study.^44^ The as‐prepared polyacrylamide‐based [EMIm]Cl/AlCl_3_ gels hold the broad‐temperature ionic conductivity with stable optically transparent features,^44^ as shown in updated Figure S4 in the Supporting Information. Therefore, such types of ionic conductive polymeric gels could be used for broad‐temperature stable transparent microwave absorption gels. In a typical example of type II ionic conductive polymeric gels, similar to the PVA‐based aqueous gels studied in the present study, they could be used to construct optically transparent–opaque switchable microwave stealth structure, in which the gels are thermally responsive as both optically switchable and microwave absorption materials. Therefore, the PVA‐based aqueous gels in the present study open a novel platform, and it is easy to manipulate the switchable temperature using different types of solvent and polymers according to the required temperature range.

Third, the smartness of optically transparent–opaque switching ability was observed. As the optically transparent gels in the structures, aqueous gels are usually sensitive temperature because temperature would change either the phase of aqueous solution in the gels or change the crystal phase in the polymers, leading to forming a thermally responsive materials in optical structures.^45^ In the present work, when the temperature was decreased to −20 °C, which is mainly due to the phase transition of the polymers. To verify the results, the transmittance at polycrystalline structures has been largely suppressed according to Figure S5 in the Supporting Information. Additionally, the impacts of the temperature change on the materials were also studied. In the determining the response time for the polymer chains in PVA, additional experiments were carried out using different types of polymers as listed in Figure S6 in the Supporting Information. Except for the pristine water, it is noted that most of the PVA‐based gels would be transformed into optically opaque within 15 s under −20 °C environment. According to the experiments for freezing a piece of ITO sheet, ITO substrate remains mechanically stable at −20 °C, as shown in Figure S7 in the Supporting Information. To clear the concerns on the polymer chains under the electromagnetic field, several points of view would be considered. Initially the major mechanism for electromagnetic attenuation in this study is mainly based on the quarter‐wavelength resonance. Furthermore, water apparently is mainly responsible for attenuating the electromagnetic waves for the much greater imaginary permittivity (from ≈13 to ≈6), while the imaginary permittivity of PVA is from ≈0.1 to ≈0.2 in the range of 12.4–18 GHz (Figure S8, Supporting Information). Based on the effective medium theory, it is suggested that water molecules are dominant role in attenuating the electromagnetic energy. According to the gel preparation process and intrinsical low power, the converted electromagnetic energy from telecommunication would contribute to heating the gels in a small limited temperature range by far. Thus, there would be very limited impacts on the polymer chains.

Furthermore, the strategies in the present study open new perspectives for potential applications. As shown in updated Figure 5c, the perspective of optically manipulatable microwave stealth structures based on ionic conductive gels suggests that four categories of optically manipulatable microwave stealth structures could be envisaged, specifically by integrating with other types of optical functional structure and devices. In Category I, the polymeric gels would be used for broad‐temperature stable optically transparent microwave stealth structures. In Category II, polymeric gels could be used to assemble into multifunctional chromatic structures integrated with other active chromatic layers (triggered with other forms of energy or stimuli),^8^ while the above microwave stealth cover remains optically transparent. In Category III, the polymeric gels could serve in the optically transparent–opaque switchable microwave stealth structure, in which the gels are thermally responsive as both optically switchable and microwave absorption materials. In Category IV, the polymeric gels could be used to assemble multifunctional structure along with another type of optically active layer (triggered with other forms of energy) for smart optical structures. In these cases, fabrication of the multilayered structure from all the categories are required additional design and fabricated using the redetermined material parameters. It is also further extended to assemble other type of optical devices and functional structures, aiming to exploring novel types of devices and structures.^46^, ^47^, ^48^, ^49^, ^50^, ^51^

Lastly, the microwave absorption ability in the present study may alleviate the potential concerns in the 5G mobile networks. According to the broadband effective absorption capabilities in the as‐fabricated sandwich structures, it is able to be used for blocking or attenuating the microwave signal in one of the branches of 5G mobile networks (24.25–40 GHz or above). Therefore, the multifunctional feature of such smart stealth windows would allow them for promoting window performance, when both optical properties and mobile networks are critical concerns. Apparently, there is plenty of room for further improving the microwave absorption capability and optically transparent–opaque switching ability. Nevertheless, the concept of design and fabrication strategies is sufficient to advance the technologies of smart windows and smart microwave absorption structures.

## 4 Conclusions

In summary, a novel optically transparent–opaque switchable smart microwave stealth structure has been demonstrated in the present work. Specifically, the aqueous gel was selected as the active materials for tailoring the complex permittivity, with purposing to achieving effective microwave absorption materials. By designing the structures with such gels, the fabricated optically transparent structures enabled to offer effective broadband microwave absorption capability in the range of 15–40 GHz. Upon cooling to −20 °C, the structure could be changed into optically opaque for the formation of polycrystal phase in the aqueous gels. The results provided a novel stage for fabricating advanced optically transparent–opaque switchable structures with capability in attenuating microwave, thus highlighting a simply strategy for achieving smart and multifunctional windows, with expectation of applying for alleviating the potential concerns in 5G mobile networks.

## Conflict of Interest

The authors declare no conflict of interest.

## Supporting information

Supporting Information
