# Supplementary material for: Ionic Conductive Gels for Optically Manipulatable Microwave Stealth Structures
Source: Adv Sci (Weinh). 2019 Nov 27;7(2):1902162. doi: 10.1002/advs.201902162 (PMC6974938; doi:10.1002/advs.201902162)
Supplement: Supplementary file 1 — Supporting Information [file ADVS-7-1902162-s001.pdf]

Copyright WILEY-VCH Verlag GmbH & Co. KGaA, 69469 Weinheim, Germany, 2019.

ADVANCED SCIENCE Open Access

Supporting Information

for *Adv. Sci.,* DOI: 10.1002/advs.201902162

Ionic Conductive Gels for Optically Manipulatable Microwave Stealth Structures

Wei-Li Song, Ya-Jing Zhang, Kai-Lun Zhang, Ke Wang, Lu Zhang, Li-Li Chen, Yixing Huang, Mingji Chen, Hongshuai Lei,* Haosen Chen,* and Daining Fang**

Supporting Information

Ionic conductive gels for optically manipulatable microwave stealth structures

Wei-Li Song, Ya-Jing Zhang, Kai-Lun Zhang, Ke Wang, Lu Zhang, Li-Li Chen, Yixing Huang, Mingji Chen, Hongshuai Lei,* Haosen Chen,* Daining Fang**

Prof. W. L. Song, Y. J. Zhang, K. L. Zhang, L. L. Chen, Dr. Y. Huang, Prof. M. Chen, Prof. H. Lei, Prof. H. Chen, Prof D. Fang
Institute of Advanced Structure Technology, Beijing Institute of Technology, Beijing, 100081, P. R. China.
E-mail: mjchen81@bit.edu.cn; lei123shuai@126.com; chenhs@bit.edu.cn; fangdn@bit.edu.cn
Prof. W. L. Song, Y. J. Zhang, K. L. Zhang, L. L. Chen, Dr. Y. Huang, Prof. M. Chen, Prof. H. Lei, Prof. H. Chen, Prof D. Fang
Beijing Key Laboratory of Lightweight Multi-functional Composite Materials and Structures, Beijing Institute of Technology, Beijing, 100081, P. R. China.
K. Wang, L. Zhang
Key Laboratory of Space Utilization, Technology and Engineering Center for space Utilization, Chinese Academy of Sciences, Beijing, 100094, China.
Y. J. Zhang, K. L. Zhang, L. L. Chen
School of Materials Science & Engineering, Beijing Institute of Technology, Beijing, 100081, P. R. China.

Experimental Section

Optically manipulatable microwave absorption materials: An optically clear gel material was prepared by crosslinking of phosphoric acid with polyvinyl alcohol. Initially, the polyvinyl alcohol (PVA) was dissolved in the water (heated at 80 °C) to prepare an aqueous polyvinyl alcohol solution. The phosphoric acid was then added into to the aqueous solution of polyvinyl alcohol of different proportions (10% by weight, 12.5% by weight, 15% by weight), followed by stirring for 1 hour at 80 °C. The mixture were then transferred into mold and cooled to room temperature to obtain electromagnetic active gels. According to different components (listed in Table 1), the prepared samples were named as 10% PVA (Sample 1), 12.5% PVA (Sample 2), 15% PVA (Sample 3), 10% PVA-5% H_3PO_4 (Sample 4), 10% PVA-12% H_3PO_4 (Sample 5), depending on the different qualities of phosphoric acid used (0% wt, 5% wt, 12% wt).

Manipulatable microwave stealth structures: There were two types of smart microwave stealth windows, i.e. single layer sandwich structure and double layer sandwich structure. For designing the multilayer structures, all the material parameters were input into the CST software, followed by sweeping the parameters for obtaining the optimized parameters. In the selection of the active PVA gels, sample 5 (10% PVA-12% H_3PO_4) was selected. According to the design of the single sandwich structure, there are four stacked parts. From the top to the bottom, they are PMMA cover (thickness: 2 mm), active PVA gel (thickness: h=4 mm), PMMA bottom (thickness: 2 mm) and ITO reflection substrate (thicker ITO layer preferred). In the design of the double layer sandwich structure, there six stacked parts. From the top to the bottom, they are PMMA cover (thickness: 2 mm), the 1st active PVA gel (thickness: H=4 mm), PET interlayer (thickness: 0.5 mm), the 2nd active PVA gel (thickness 3 mm), PMMA bottom (thickness: 2 mm) and ITO reflection substrate (thickness: 0.5 mm).

Material characterizations: In the measurement of the viscosity, the digital viscometer (Ni Run DV-2+PRO) was used. The optical properties of the gels were obtained on an ultraviolet-visible spectrophotometer.

Measurement of complex permittivity: An Ku-band waveguide method was chosen for measuring complex permittivity of pure H_2O, 10% PVA 10% PVA-5% H_3PO_4, 10% PVA-8% H_3PO_4, 10% PVA-10% H_3PO_4, 10% PVA-12% H_3PO_4. The as-fabricated samples were cut into matched size with rectangular waveguide of Ku-band (7.75 mm×15.7 mm). Then, the prepared samples were put into measuring waveguide, which was connected to a Keysight E5071C vector network analyzer.

Calculated EM absorption performance from complex permittivity: The as-measured complex permittivity are used for calculating the absorption properties of materials with different thicknesses. Typically, the RL can be expressed as:

$$Z_{in} = \sqrt{\frac{\mu_r}{\varepsilon_r}} \tanh\left[j\frac{2\pi}{c}\sqrt{\mu_r \varepsilon_r}\, fd \right], \tag{1}$$

$$RL=20\log\frac{|Z_{in}-1|}{|Z_{in}+1|}, \tag{2}$$

where Z_{in} is the normalized input impedance, c the light velocity, f the frequency, ε_r the complex permittivity, and μ_r the complex permeability.

Arch method for experimental measurement of microwave absorption performance: In the measurement of practical absorption performance, the fabricated multilayer windows (with planar size of 180 × 180 mm^2) were placed on the metal plate of arch setup. The transmit and receive antennas were connected by a vector network analyzer (Aglient Technology NS5255A).

Figure S1. Complex permittivity of various gel samples with different PVA contents: (a) S1 (0% PVA), (b) S2 (10% PVA), (c) S3 (12.5% PVA), (d) S4 (15% PVA).

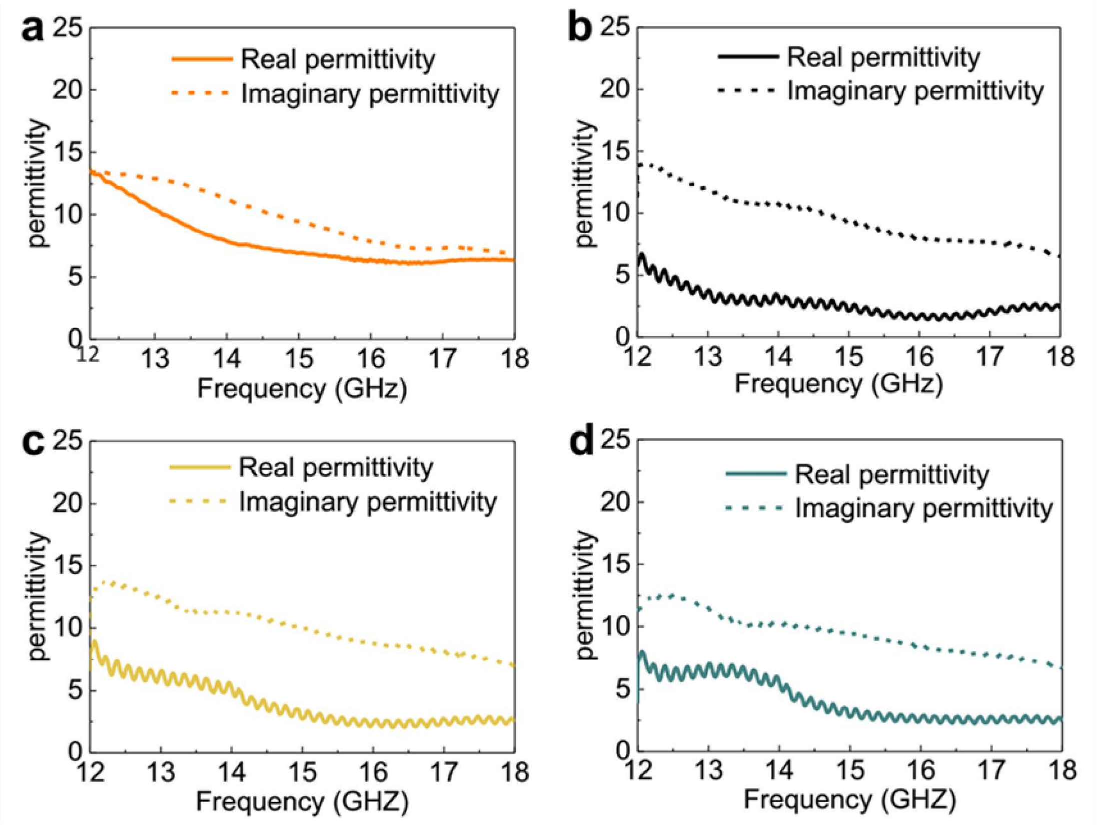

Figure S2. Calculated microwave absorption performance of various gel samples with different component contents: (a) S1 (0% PVA), (b) S2 (10% PVA), (c) S3 (10% PVA-5% H_3PO_4), (d) S4 (10% PVA-8% H_3PO_4), (e) S5 (10% PVA-12% H_3PO_4).

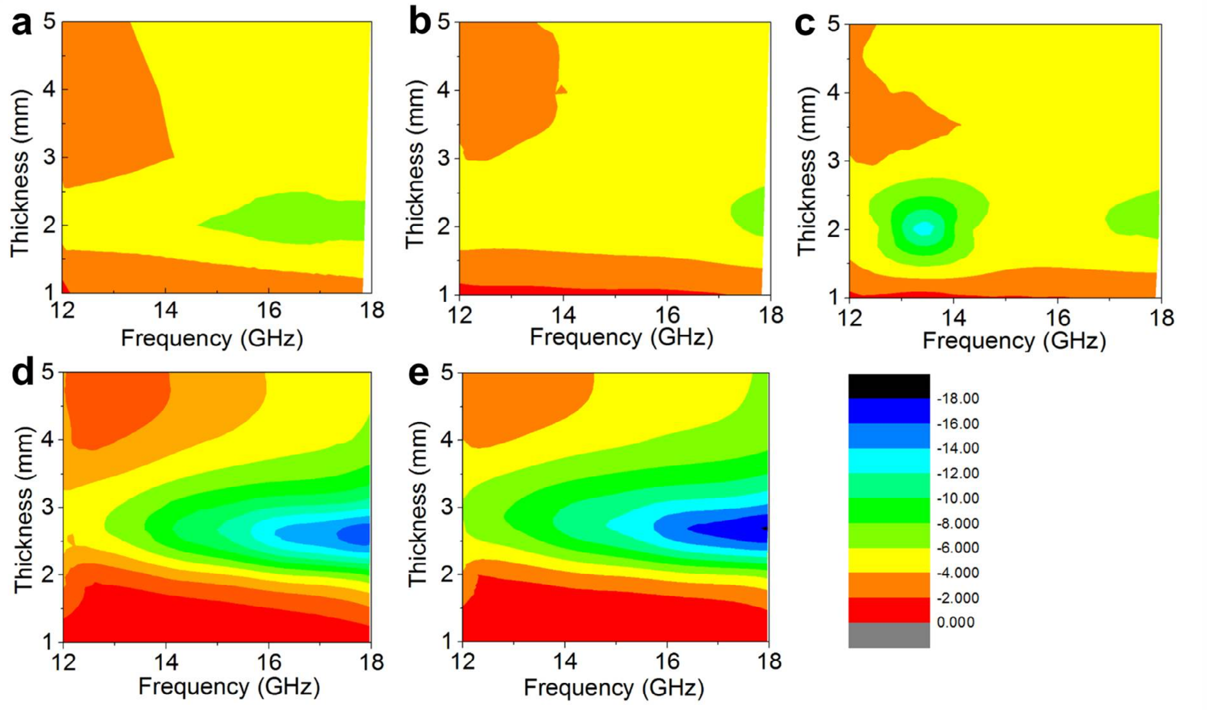

Figure S3. Experimental measured microwave absorption performance (TM mode) by the arch method: the single layer sandwich structure at (a) room temperature and (b) -20□; the double layer sandwich structure at (c) room temperature and (d) -20□. The incident angles were set at 5°, 30° and 45°.

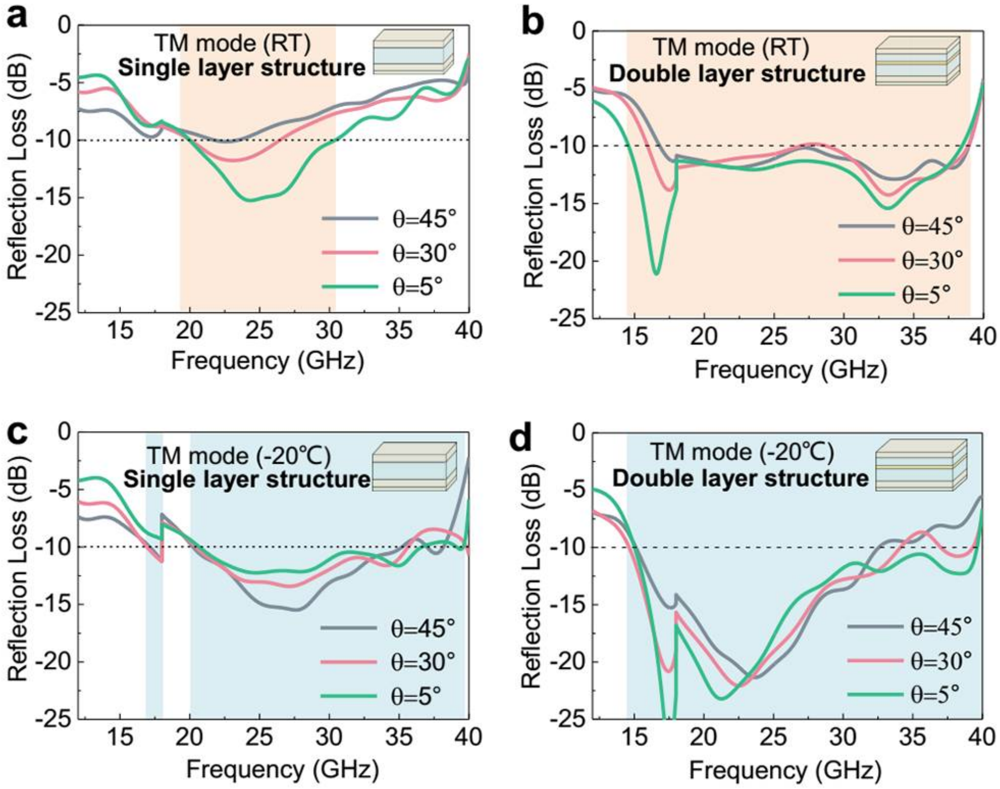

Figure S4 The as-prepared transparent polyacrylamide-based [EMIm]Cl/$AlCl_3$ gels

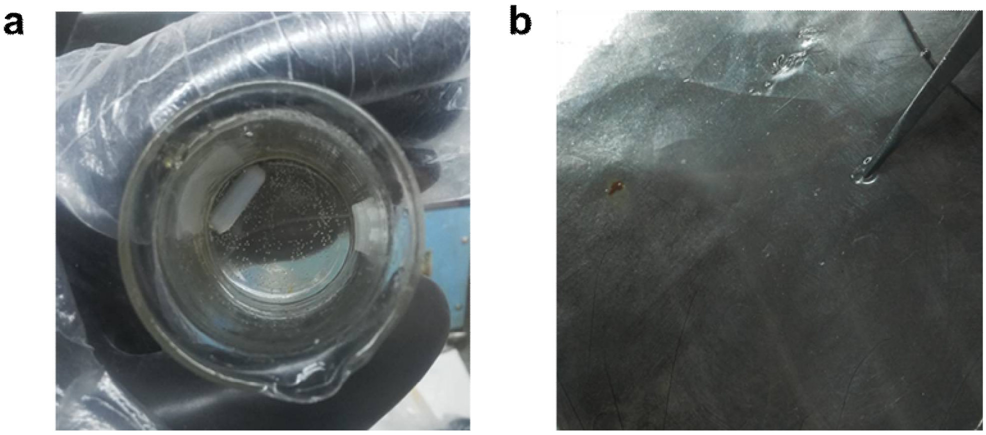

Figure S5 The UV-vis spectrum of the PVA-based gels at -20 °C

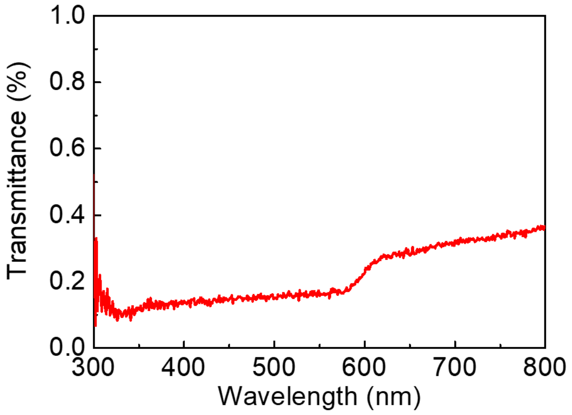

Figure S6 The estimated phase-transition time of various gels under -20 ^{o}C.

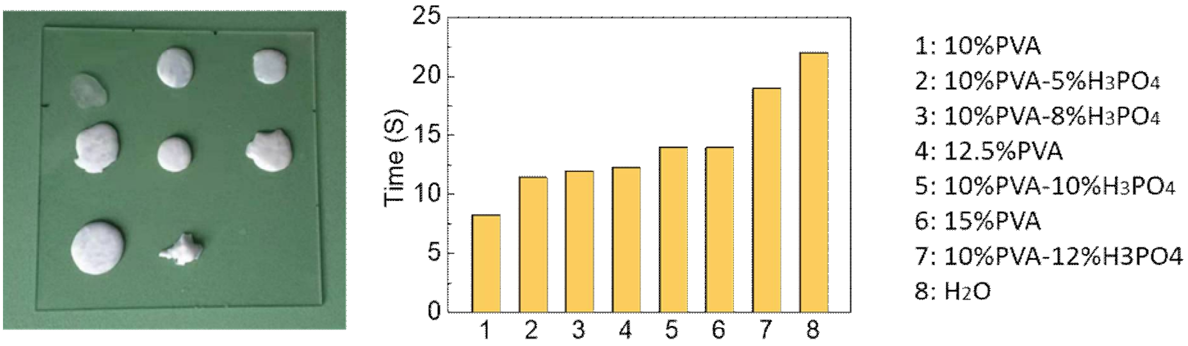

Figure S7 (a) The experimental measurement of the structure under -20 °C; Comparison of the mechanical stability for the ITO-based polymer film (b) at room temperature and (c) at -20 °C.

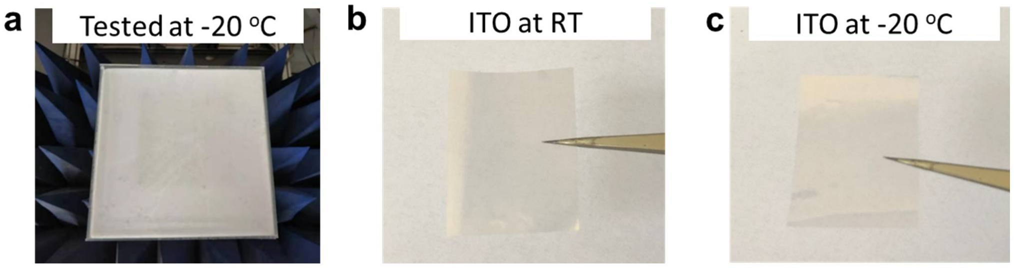

Figure S8 The complex permittivity of the pristine PVA

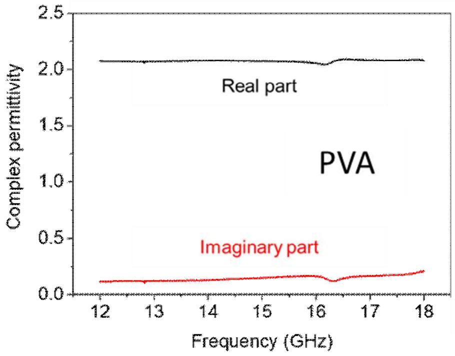

Table S1 Typical smart optical windows with microwave absorption and manipulatable optical functions. Symbol "—" indicates the terms that have not been concerned or targeted in the study.

Smart optical structures or devices for smart windows	Microwave absorption function				Optical function manipulation			Refs
	Initial effective absorption range (GHz)	Manipulated absorption effective range (GHz)	Covering a branch of 5th G network frequency	Mechanism for microwave absorption	Initial optical properties	Manipulated optical properties	Mechanism for manipulating optical function	
Sandwich structures with single layer aqueous gels	20~30 GHz	21~40 GHz	Partial	Optical transparent polar gels for absorbing microwave	Optially transparent	Optially opaque (white)	Reversible crystalline transition of gels	This work
Sandwich structures with double-layer aqueous gels	12.4~40 GHz	15~40 GHz	Yes	Optical transparent polar gels for absorbing microwave	Optially transparent	Optially opaque (white)	Reversible crystalline transition of gels	This work
		—	Not targeted		Optially transparent	—	—	S1
		—	Not targeted		Optially transparent	—	—	S2
		—	Not targeted		Optially transparent	—	—	S3
		—	Not targeted		Optially transparent	—	—	S4
	—	—	Not targeted	—	Optially transparent	Optially opaque (black)	Redox reaction	S5
	—	—	Not targeted	—	Optially transparent (green)	Optially transparent (blue)	Redox reaction	S6
	—	—	Not targeted	—	Optially transparent	Optially opaque (black)	Redox reaction	S7
	—	—	Not targeted	—	Optially transparent	Optially opaque (black)	Redox reaction	S8
	—	—	Not targeted	—	Optially transparent	Optially opaque (brawn)	Redox reaction	S9
	—	—	Not targeted	—	Optially opaque (white gary)	Optially opaque (black)	Redox reaction	S10
	—	—	Not targeted	—	Optially opaque (cyan)	Optially opaque (yellow)	Redox reaction	S11
	—	—	Not targeted	—	Optially transparent	Optially opaque (black)	Redox reaction	S12
	—	—	Not targeted	—	Optially transparent	Optially opaque (black)	Redox reaction	S13
	—	—	Not targeted	—	Optially transparent	Optially opaque (white)	Adsorption	S14

References:

[S1] T. Jang, H. Youn, Y. Shin, L. Guo, *ACS Photonics*. **2014**, *1*, 279.

[S2] C. Zhang, Q. Cheng, J. Yang, J Zhao, T. Cui, *Appl. Phys. Lett.* **2017**, *110*, 143511.

[S3] K. Chen, L. Cui, Y. Feng, J Zhao, T. Jing, B. Zhu, *Opt. Express* **2017**, *5*, 5572.

[S4] I. Lee, S. Yoon, J. Lee, I. Hong, *Electron. Lett.* **2016**, *7*, 555.

[S5] G. Cai, X. Wang, M. Cui, P. Darmawan, J. Wang, A. L. S. Eh, P. S. Lee, *Nano Energy* **2015,** *12*, 258.

[S6] K. Wang, H. Wu, Y. Meng, Y. Zhang, Z. Wei, *Energy Environ. Sci.* **2012,** *5*, 8384.

[S7] L. Shao, J. W. Jeon, J. L. Lutkenhaus, *Chem. Mater.* **2012,** *24*, 181.

[S8] S. Cong, Y. Tian, Q. Li, Z. Zhao, F. Geng, *Adv. Mater.* **2014,** *26*, 4260.

[S9] A. Llorde ´s, G. Garcia, J. Gazquez, D. Milliron, *Nature* **2013,** *15*, 323.

[S10] W. Bao, J. Wan, X. Han, X. Cai, H. Zhu, D. Kim, D. Ma, Y. Xu, J. N. Munday, H. D. Drew, M. S. Fuhrer, L. Hu, *Nat. Commun.* **2014,** *5*, 4224.

[S11] M. R. J. Scherer, L. Li, P. M. S. Cunha, O. A. Scherman, U. Steiner, *Adv. Mater.* **2012,** *24*, 1217.

[S12] P. Yang, P. Sun, Z. Chai, L. Huang, X. Cai, S. Tan, J. Song, W. Mai, *Angew. Chem. Int. Ed.* **2014,** *53*, 11935.

[S13] A. M. Österholm, D. E. Shen, J. A. Kerszlis, R. H. Bulloch, M. Kuepfert, A. L. Dyer, J. R. Reynolds, *ACS Appl. Mater. Interfaces* **2015,** *7*, 1413.

[S14] R. T. Wen, C. G. Granqvist, G. A. Niklasson, *Adv. Funct. Mater.* **2015,** *25*, 3359.
